# PROTAC mediated FKBP12 degradation enhances Hepcidin expression via BMP signaling without immunosuppression activity

**DOI:** 10.1038/s41392-022-00970-8

**Published:** 2022-05-27

**Authors:** Tianbai Zhong, Xiuyun Sun, Li Yu, Yongbo Liu, Xin Lin, Yu Rao, Wei Wu

**Affiliations:** 1 MOE Key Laboratory of Protein Sciences, Beijing Advanced Innovation Center for Structural Biology, School of Life Sciences, Tsinghua University, Beijing, 100084 P. R. China; 2 MOE Key Laboratory of Protein Sciences, School of Pharmaceutical Sciences, MOE Key Laboratory of Bioorganic Phosphorus Chemistry & Chemical Biology, Tsinghua University, Beijing, 100084 China; 3 Institute for Immunology and School of Medicine, Tsinghua University, Beijing, 100084 China

**Keywords:** Chemical biology, Metabolic disorders

**Dear Editor**,

Hepcidin is a 25-amino acid peptide acting as a pivotal negative regulator in iron homeostasis, which can bind to an iron exporter, ferroportin 1, and induce its internalization and degradation.^1^ Hepcidin is produced in hepatocytes mainly under the control of BMP signaling. BMP2/6, secreted by liver endothelial cells in response to iron level, binds to BMP type I and type II receptors and triggers the phosphorylation of Smad1/5/8 which directly promotes hepcidin expression.^1^ The immunophilin family protein FKBP12 is associated with BMP type I receptors to prevent uncontrolled receptor activation.^2^ A previous study revealed FKBP12 ligands FK506 and Rapamycin can release FKBP12 from BMP type I receptors to activate BMP signaling and hepcidin expression.^2^ Other groups also demonstrated that FK506-activated BMP signaling accelerated the wound healing process or inhibited cancer metastasis. However, by binding to FKBP12, FK506, and Rapamycin potently inhibit the activities of Calcineurin or mTOR, respectively, and function as immunosuppression reagents in the clinic.^3^ This makes FK506 and Rapamycin unlikely useful for hepcidin regulation in the clinic. Proteolysis-targeting chimera (PROTAC) is an emerging chemical approach capable of degrading target proteins through a ubiquitin-proteasome system.^4^ Several PROTAC molecules targeting FKBP12 were developed using various FKBP12 ligands,^4^ RC32 was developed by linking Rapamycin with Pomalidomide and proved highly potent and applicable in vivo.^5^ We, therefore, testified RC32 for hepcidin regulation in vitro and in vivo. Our results revealed that PROTAC-mediated FKBP12 degradation is an ideal strategy to upregulate hepcidin expression without immunosuppression activity.

We first characterized the efficiency of RC32-induced FKBP12 degradation in hepatocellular carcinoma (HCC) cell lines. RC32 efficiently induced FKBP12 degradation in Hep3B and HuH7 with DC_50_ values at 0.9 and 0.4 nM, respectively (Supplementary Fig. 1a, b). RC32-induced FKBP12 protein degradation was quite fast since almost complete FKBP12 degradation was achieved in 4 to 6 h (Supplementary Fig. 1c). Consistent with the previous report,^5^ RC32-promoted FKBP12 degradation was rather specific since at low concentrations, only FKBP12 was affected, among several other FKBP proteins closely related to FKBP12 (Supplementary Fig. 1d).

Knowing RC32 is a potent degrader of FKBP12 in HCC cell lines, we explored whether RC32 could activate BMP signaling similar to FK506 and Rapamycin.^2^ As expected, treatment of Hep3B or Huh7 cells with RC32 for 15 h induced Smad1/5/8 phosphorylation in a dose-dependent manner (Fig. 1a and Supplementary Fig. 2a). The BMP target genes, *ID1*, *SKIL*, *SMAD7*, were also upregulated in Hep3B and HuH7 cells upon treatment (Supplementary Fig. 2b). Careful time course experiments indicated that the kinetics of Smad1/5/8 phosphorylation induced by RC32, FK506, or Rapamycin was largely similar (Supplementary Fig. 2c). Yet, a dramatic difference was observed in washout experiments. RC32-induced Smad1/5/8 phosphorylation lasted for more than 36 h, due to slow recovery of FKBP12 proteins, which is consistent with the previous report,^5^ whereas the p-Smad1/5/8 signal dropped to basal level in less than 4 h after removal of FK506 or Rapamycin (Fig. 1b).

**Fig. 1:**
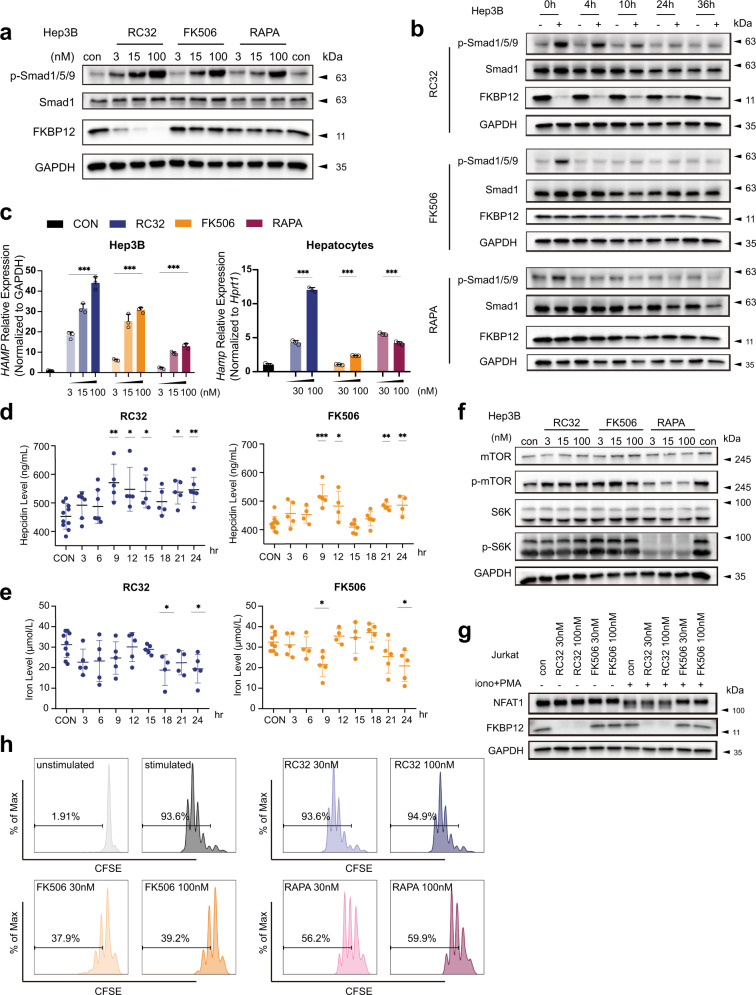
RC32 elevates hepcidin expression without immunosuppression. **a** RC32 induced BMP signaling activation. Hep3B cells were treated with 3, 15, 100 nM of RC32, FK506, or Rapamycin for 15 h. BMP signaling was verified by phosphorylation of Smad1 (Ser463/465)/Smad5 (Ser463/465)/Smad8 (Ser465/467). Total Smad1 and GAPDH were used as loading controls. **b** Duration of BMP activation after RC32, FK506, or Rapamycin treatment. Hep3B cells were treated with 15 nM drugs for 15 h and then further cultured without drugs for indicated time periods. BMP signaling and FKBP12 protein levels were analyzed by Western Blots. **c** RC32 promoted hepcidin expression in Hep3B and mice primary hepatocytes. Cells were treated with drugs as indicated concentrations for 15 h and then collected for RT-qPCR analysis of hepcidin (HAMP) expression. Primary hepatocytes isolated from mice were seeded in serum-free medium overnight and then treated with drugs for 15 h in hepatocyte culture medium. The results are presented as the mean ± SD, *n* = 3. one-way analysis of variance (ANOVA), *p < 0.05, **p ≤ 0.01, ***p ≤ 0.001. **d** Serum Hepcidin levels and **e** Serum Iron levels after RC32/FK506 treatment in Mice. The results are presented as the mean ± SD, *n* = 4–6. One-way analysis of variance (ANOVA), *p < 0.05, **p ≤ 0.01, ***p ≤ 0.001. **f** RC32 did not inhibit mTOR activity in hepatocellular carcinoma cells. Hep3B were treated with drugs as indicated concentrations for 15 h and then harvested for Western Blotting analysis. mTOR activity was verified by phosphorylation of mTOR (Ser2448, p-mTOR) and phosphorylation of S6 Kinase (Thr389, p-S6K). Total mTOR, S6 Kinase (S6K), and GAPDH were used as loading controls. **g** RC32 did not inhibit Calcineurin. Jurkat cells were pretreated with RC32 or FK506 for 4 h and then stimulated with ionomycin (1 μg/mL) and PMA (20 ng/mL) for 30 min followed by Western Blot assay testing NFAT1 dephosphorylation. GAPDH served as a loading control. **h** RC32 did not inhibit in vitro stimulated PBMC proliferation. Peripheral blood mononuclear cells (PBMC) were stained with CFSE and stimulated with anti-CD3/anti-CD28 antibodies together with indicated drugs. Three days after stimulation, cells were collected and stained with APC anti-human CD3 antibody and then followed by Flow cytometry analysis. PBMCs from two donors were used in two independent experiments and similar results were obtained

Next, we verified whether RC32 has the ability to upregulate the expression of the hepcidin gene. Hepcidin mRNA (*HAMP*) levels were significantly increased in Hep3B and HuH7 cells in response to RC32 treatment for 15 h, similar to FK506 or Rapamycin treatment (Fig. 1c and Supplementary Fig. 2d). A significant upregulation of hepcidin expression was also detected in cultured primary hepatocytes isolated from mice (Fig. 1c). Consistent with the sustained Smad1/5/8 phosphorylation (Fig. 1b), RC32-induced Hepcidin expression declined slowly after RC32 removal, whereas the induction by FK506 or Rapamycin dropped quickly (Supplementary Fig. 2e).

Furthermore, we explored whether RC32 can upregulate hepcidin expression in mice. As indicated in Supplementary Fig. 3a, RC32 or FK506 was injected in male mice at 0 and 12 h, and blood samples were collected at 3, 6, 9, 12, 15, 18, 21, and 24 h to monitor Hepcidin and iron levels in serum. Consistent with the previous report,^5^ FKBP12 protein was completely degraded in liver samples 12 h after RC32 application (Supplementary Fig. 3b). Serum Hepcidin levels were indeed elevated after RC32 or FK506 injection (Fig. 1d) and accordingly, serum iron levels were reduced by both drugs (Fig. 1e). The results shown in Fig. 1d seem to suggest a persistent enhancement of hepcidin expression by RC32 and a relatively transient upregulation by FK506. This is consistent with their different capacity to regulate Smad phosphorylation and hepcidin expression (Fig. 1b and Supplementary Fig. 2e), though, the pharmaceutical kinetics difference of the two drugs was not clear. Together, these results confirmed that RC32, an FKBP12 degrader, can regulate hepcidin expression at least as good as FK506, both in vitro and in vivo.

Hepcidin expression could also be upregulated *via* JAK/STAT3 pathway by inflammatory cytokines such as IL-6.^1^ We observed no significant change of phosphorylated STAT3 (Tyr705) after RC32, FK506, or Rapamycin treatment in HCCs (Supplementary Fig. 3c), suggested that hepcidin activation by FKBP12 degradation or releasing is not attributed to JAK/STAT3 signaling. Furthermore, DMH1 and LDN212854, two inhibitors of the type I BMP receptor ALK2, dramatically inhibited the upregulation of hepcidin and *ID1*, another BMP target, by RC32, FK506, or Rapamycin treatment (Supplementary Fig. 3d). These results further confirmed that RC32 functioned through BMP signaling activation.

The results above clearly demonstrated that, by degrading FKBP12, RC32 can induce hepcidin expression, as good as FK506 or Rapamycin. Next, we verified whether RC32 had similar immunosuppression activity as FK506 or Rapamycin. Rapamycin, a potent mTOR inhibitor, reduces mTOR phosphorylation and subsequent S6K phosphorylation, leading to severe proliferation inhibition and autophagy activation. In agreement with the previous report,^5^ we observed no inhibition of mTOR or S6K phosphorylation in Hep3B and HuH7 cells treated with RC32 or FK506 (Fig. 1f and Supplementary Fig. 4a). Consistently, cell proliferation was not affected by RC32 treatment (Supplementary Fig. 4b). We also monitored autophagy induction in an LC3 reporter NRK cell line by counting the puncta fluorescence signal, a characteristic feature of acute Rapamycin treatment. No more LC3 puncta was induced by RC32 treatment, whereas Rapamycin caused massive puncta induction (Supplementary Fig. 4c, d). In this NRK cell line, Rapamycin inhibited mTOR and S6K phosphorylation and cell proliferation, while RC32 did none of them, but significant FKBP12 elimination (Supplementary Fig. 4e, f). The above results indicated that sharply in contrast with Rapamycin, RC32 does not inhibit mTOR.

FK506 blocks the phosphatase activity of Calcineurin by binding to FKBP12 and achieves immunosuppression through this mechanism.^3^ During T cell activation, transcription factor NFAT is dephosphorylated by Calcineurin and then translocates into the nucleus to drive the expression of T cell activation-related genes such as IL-2. We stimulated Jurkat cell by ionomycin/PMA treatment and observed NFAT1 dephosphorylation (Fig. 1g), IL-2 mRNA expression, and IL-2 protein accumulation in the medium (Supplementary Fig. 4g, h). FK506 strongly inhibited these activities but RC32 showed no effect. These results indicated that in contrast to FK506, RC32 does not inhibit Calcineurin.

To further confirm that RC32 does not possess immunosuppression activity, we utilized the in vitro PBMC (peripheral blood mononuclear cells) stimulation assay to compare these three drugs. PBMCs were stimulated by anti-CD3 and anti-CD28 antibodies to obtain massive proliferation in vitro. Both FK506 and Rapamycin exhibited remarkable inhibition of T cell expansion and relevant cytokines secretion, RC32 revealed no inhibition at all (Fig. 1h and Supplementary Fig. 5a–d). FKBP12 was significantly degraded in PBMC (Supplementary Fig. 5e). Taken together, these results clearly demonstrated that, unlike FK506 or Rapamycin, RC32 has no immunosuppression activity.

In this study, using a PROTAC molecule RC32 to promote FKBP12 degradation, we achieved BMP signaling activation and hepcidin upregulation as good as using classical FKBP12 binding molecules FK506 and Rapamycin in vitro. In mice, RC32 transiently elevated serum hepcidin and reduced serum iron levels, in a manner comparable to FK506. Compared to FK506 or Rapamycin with instinct side-effects, at least in vitro, RC32 does not inhibit mTOR or Calcineurin and shows no immunosuppression activity. Derivatives of FKBP12 binding molecules that lack immunosuppression activity were developed and their capacity in hepcidin regulation should also be tested in the future. Our study, therefore, suggested that PROTAC-mediated FKBP12 degradation could be a novel and safe approach to treat iron overload diseases resulting from low hepcidin.

FKBP12 associates with the BMP receptor and prevents uncontrolled BMP signaling activation. Activation of BMP signaling by releasing FKBP12 has significant applications in BMP signaling deficiency-related diseases such as Idiopathic pulmonary arterial hypertension (IPAH),^2^ wound healing, or cancer metastasis. In all these cases, PROTAC-mediated FKBP12 degradation could serve as an alternative approach.

## Supplementary information

Supplemental Information

## Data Availability

The data are available from the corresponding author on reasonable request.
