# Correction: PROTAC mediated FKBP12 degradation enhances Hepcidin expression via BMP signaling without immunosuppression activity

**DOI:** 10.1038/s41392-022-01051-6

**Published:** 2022-06-22

**Authors:** Tianbai Zhong, Xiuyun Sun, Li Yu, Yongbo Liu, Xin Lin, Yu Rao, Wei Wu

**Affiliations:** 1 MOE Key Laboratory of Protein Sciences, Beijing Advanced Innovation Center for Structural Biology, School of Life Sciences, Tsinghua University, 100084 Beijing, China; 2 MOE Key Laboratory of Protein Sciences, School of Pharmaceutical Sciences, MOE Key Laboratory of Bioorganic Phosphorus Chemistry & Chemical Biology, Tsinghua University, 100084 Beijing, China; 3 Institute for Immunology and School of Medicine, Tsinghua University, 100084 Beijing, China

**Keywords:** Chemical biology, Metabolic disorders

Correction to: *Signal Transduction and Targeted Therapy* 10.1038/s41392-022-00970-8, published online 27 May 2022

In this article, the funding from “National Natural Science Foundation of China to YR (Grant# 82125034), National Key R&D Program of China (#2020YFE0202200, #2021YFA1300200).” was omitted.

The original article has been corrected.

