# Supplementary material for: PROTAC mediated FKBP12 degradation enhances Hepcidin expression via BMP signaling without immunosuppression activity
Source: Signal Transduct Target Ther. 2022 May 27;7:163. doi: 10.1038/s41392-022-00970-8 (PMC9135734; doi:10.1038/s41392-022-00970-8)
Supplement: Supplementary file 1 — Supplemental Information [file 41392_2022_970_MOESM1_ESM.pdf]

Supplementary materials for

PROTAC mediated FKBP12 degradation enhances Hepcidin expression via BMP signaling without immunosuppression activity

Tianbai Zhong[1]; Xiuyun Sun[2]; Li Yu[3]; Yongbo Liu[2]; Xin Lin[3]; Yu Rao[2]*; Wei Wu[1]*

[1]MOE Key Laboratory of Protein Sciences, Beijing Advanced Innovation Center for Structural Biology, School of Life Sciences, Tsinghua University, Beijing 100084, China

[2]MOE Key Laboratory of Protein Sciences, School of Pharmaceutical Sciences, MOE Key Laboratory of Bioorganic Phosphorus Chemistry & Chemical Biology, Tsinghua University, Beijing 100084, China.

[3]Institute for Immunology and School of Medicine, Tsinghua University, Beijing 100084, China.

*Corresponding authors. Wei Wu: wwu@mail.tsinghua.edu.cn, Yu Rao: yrao@mail.tsinghua.edu.cn.

This PDF files includes

Methods and Materials

Supplementary Table 1

Supplementary Table 2

Supplementary Figure 1

Supplementary Figure 2

Supplementary Figure 3

Supplementary Figure 4

Supplementary Figure 5

Materials and Methods

Cells Lines, Culture Condition and Treatment

Hep3B, Huh7 and Jurkat cells were purchased from cell bank (Shanghai) of Chinese Academy of Sciences (www. Cellbank.org.cn). NRK-LC3-CFP reporter line were provided by Yu Li lab as described in[1]. Hep3B were maintained in MEM (Gibco) supplement with 15% FBS (AusGeneX) and 1% Penicillin-Streptomycin (Macgene), L-glutamine (beyotime), NEAA (Gibco). Huh7 were maintained in DMEM (Gibco) supplement with 10% FBS and 1% Penicillin-Streptomycin and L-glutamine. Jurkat were maintained in RPMI-1640 (Gibco) supplement with 10% FBS and 1% Penicillin-Streptomycin. NRK-LC3-CFP reporter cell line was maintained in DMEM supplement with 10% FBS and 1% Penicillin-Streptomycin. Mice Primary Heptocytes were isolated as described[2] and cultured in hepatocyte culture medium (Procell CM-M033). All cells were cultured in 37°C incubator with 5% CO2 condition. RC32 were synthesized as described[3]. FK506 (Tacrolimus), Rapamycin, pomalidomide, Bortezomib, Carfilzomib, DMH1, LDN212854 were purchased from Selleck. Ionomycin and PMA were purchased from Beyotime. All treatment were performed under complete medium if not mentioned.

Antibodies

All antibodies used in this study were purchased from commercial companies and the detail information are listed in the Supplementary table 1.

Mice Treatment

Wild-type (WT) C57BL/6N male mice (8 weeks) were from Beijing Vital River Laboratory Animal Technology. Animal experiment was approved by institutional Animal Care and Use Committee of Tsinghua University (AP-WW-18-1). For FK506 treatment, mice were treated with FK506 (10 mg/kg) or dissolvent at 0 and 12 h by subcutaneous injection. Vehicle treated mice were euthanized at 3 and 15 hr post-injection and FK506 treated mice were euthanized at 3, 6, 9, 12, 15, 18 and 24 hr post-injection. FK506 were dissolved in corn oil with 5% DMSO and corn oil with 5% DMSO was used as control. For RC32 treatment. mice were treated with RC32 (30 mg/kg) or dissolvent at 0 and 12 hr by intraperitoneal injections and analyzed as the FK506 group. RC32 were dissolved in 0.9% NaCl (aq.) containing 5% DMSO and 10% castor oil and the dissolvent was used as control. Blood samples were collected after anesthetized and then mice euthanized. Liver tissues for Western Blot were collected immediately after euthanized.

Western Blot Assay

Cells were seeded in 12-well plates. Compounds were dissolved in DMSO than diluted in medium at suitable concentration such that the final concentration of DMSO is not more than 0.2%. After treatment, cells are washed by PBS, then lysed by 1x loading buffer contain PMSF, protease inhibitor cocktail and phosphatase inhibitor cocktail (biomake) on ice for 10 min. The samples were collected and heated at 95 ℃ for 10 min. Tissue samples (about 10-20 mg) were collected and homogenized in RIPA buffer (beyotime) containing PMSF, protease inhibitor cocktail and phosphatase inhibitor cocktail and then lysed on ice for 20 min. The lysates were cleared by centrifugation for 20 min at 4 °C, and supernatants collected for further analysis. Samples were separated in 8% -15% SDS-PAGE

and transferred to PVDF membranes (Merck Millipore). The membranes were placed in 5% no-fat milk and incubated with corresponding antibody (refer to supplementary table 1 for details) at 4 °C overnight followed by relevant HRP secondary-antibodies. The membranes were visualized by StarSignal Chemiluminescent Assay Kit (GenStar) and MiniChemi.

In Vitro PBMC Stimulation Assay

Human peripheral blood mononuclear cells (PBMCs) from healthy donors were provided by Department of Hematology, Peking University People's Hospital under an institutional review board–approved protocol. PBMCs were cultured in RPMI-1640 media supplement with 20% FBS and 1% Penicillin-Streptomycin, Stimulation was performed in media with 10% FBS. At the day 0, PBMC were recovery in RPMI-1640 medium supplemented with 10% FBS and IL-2 (200 IU/ml) overnight. 48 well plates were coating with anti-CD3 (5 μg/ml; BioLegend, #317315), anti-CD28 (1 μg/ml; BD Biosciences, #555725), and human fibronectin (5 μg/ml; BD Biosciences, #354008) at 4 °C overnight. At day 1 PBMC were stained with 5 μM PMSF (eBioscience™), then seeded in coated plates in RPMI-1640 medium supplemented with 20% FBS and IL-2 (200 IU/ml). RC32, FK506 or rapamycin was added for treatment. At day 3, cells were collected and stained with APC anti-human CD3 Antibody (1：200; BioLegend, #317315) and fixed with 2% PFA. Flow cytometry data were acquired by BD Fortessa.

Enzyme-linked immunosorbent assay

The cytokines released into culture medium after PBMC stimulation were measured using human IL-2 (eBioscience, #88-7025-88), human IFN-γ (eBioscience, #88-7316-88), and human TNF-α (eBioscience, #88-7346-88) enzyme-linked immunosorbent assay kits as manufacturer's instructions. The cytokines released by Jurkat cell stimulated with PMA and ionomycin were measured using Human IL-2 ELISA Kit (PI580) as manufacturer's instructions.

Quantitative Real-time RT-PCR

Total RNA were extracted using EZ-10 DNAaway RNA Mini-Preps Kit (Sangen Biotech). cDNA were synthesized by RevertAid First Strand cDNA Synthesis Kit (Thermo Scientific). Gene expression level were measured using ChamQ SYBR qPCR Master Mix (Vazyme) and reactions performed with CFX96 Real-Time System (BIO-RAD) or Roche480 system (Roche). Expression values were normalized to GAPDH expression. The PCR primer sequences are shown in Supplementary table 2.

Cell Proliferation Assay

500-1500 Cells were seeded in 96-well plate in triplicates. Drug were introduced in wells 24 hrs later at indicated concentration. The DMSO concentration are not more than 0.1% in the medium. The cells were kept in cell incubator for 72 hours. After that, 10 μL CCK-8 (Vazyme) were added in each well. The absorbance was detected at 450 nm by SpectraMax Plus Microplate Reader after incubating for 2-6 hours.

Serum hepcidin and Iron Quantification

Serum hepcidin level were measured using Murine Hepcidin enzyme linked immunosorbent assay kit (MEIMIAN #MM-44770), according to the manufacturer's protocol and calculated according to the standard curve using GraphPad Prism V8. Serum Iron concentration were measured using Micro Serum Iron Concentration Assay Kit (Solarbio Life sciences) according to the manufacturer's protocol.

Live-Cell Imaging

NRK-LC3-CFP reporter cell line was kindly provided by Yu Li Lab. Cells were seeded in 35 mm Glass bottom dishes (Cellvis) and drugs were added in the medium 3 hrs later. Images were acquired 15 hrs later using Zeiss LSM980 Airyscan. Images were analysed by Imaris and GraphPad Prism V8.

Statistical Analysis

Data shown were compared by one-way analysis of variance (ANOVA) or two-tailed unpaired Student's t test using GraphPad Prism V8.

References

1 Rong, Y. *et al.* Clathrin and phosphatidylinositol-4,5-bisphosphate regulate autophagic lysosome reformation. *Nat Cell Biol* **14**, 924-934, doi:10.1038/ncb2557 (2012).

2 Han, J. *et al.* The CREB coactivator CRTC2 controls hepatic lipid metabolism by regulating SREBP1. *Nature* **524**, 243-246, doi:10.1038/nature14557 (2015).

3 Sun, X. *et al.* A chemical approach for global protein knockdown from mice to non-human primates. *Cell Discov* **5**, 10, doi:10.1038/s41421-018-0079-1 (2019).

Supplementary Tables

Supplementary Table 1

Antibodies

**Western Blot**			
Protein	Antibody	Brand	Cat. Number
FKBP12	Anti-FKBP12 Antibody (H-5)	Santa Cruz	sc-133067
GAPDH	GAPDH-HRP mouse monoclonal antibody	SUNGENE BIOTECH	LK9002T
Smad1	Smad1 (D59D7) XP® Rabbit mAb	CST	#6944
p-Smad1/5/9	Phospho-Smad1 (Ser463/465)/ Smad5 (Ser463/465)/ Smad9 (Ser465/467) (D5B10) Rabbit mAb	CST	#13820
mTOR	mTOR (7C10) Rabbit mAb	CST	#2983
p-mTOR	Phospho-mTOR (Ser2448) (D9C2) XP® Rabbit mAb	CST	#5536
S6K	p70 S6 Kinase Antibody	CST	#9202
p-S6K	Phospho-p70 S6 Kinase (Thr389) Antibody	CST	#9205
NFAT1	NFAT1 (D43B1) XP® Rabbit mAb	CST	#5861
FKBP52	Anti-FKBP52 antibody [EPR21125]	abcam	ab230951

FKBP51	Anti-FKBP51 antibody [EPR6617]	abcam	ab126715
FKBP38	Anti-FKBP38 antibody [EPR7441(2)]	abcam	ab129113
STAT3	Stat3 (124H6) Mouse mAb	CST	#9139
p-STAT3	Phospho-Stat3 (Tyr705) (D3A7) XP® Rabbit mAb	CST	#9145
Smad2/3	Smad2/3 Antibody	CST	#3102
p-Smad2/3	Phospho-Smad2 (Ser465/467)/Smad3 (Ser423/425) (D27F4) Rabbit mAb	CST	#8828

PBMC stimulation

Protein	Antibody	Brand	Cat. Number
CD3	Ultra-LEAF™ Purified anti-human CD3 Antibody	Biolegend	317325
CD28	BD Pharmingen™ Purified NA/LE Mouse Anti-Human CD28	BD bioscience	555725
CD3	APC anti-human CD3 Antibody	Biolegend	344812

Supplementary Table 2

primers for qPCR

	Gene	sequence(5'-3')
Human	*GAPDH*	FW:GTCAAGGCTGAGAACGGGAAGC
		REV:GGACTCCACGACGTACTCAGCG

	HAMP	FW:CAGCTGGATGCCCATGTTC
		REV:CAGCAGCCGCAGCAGAA
	ID1	FW:AAACGTGCTGCTCTACGACA
		REV:GGGGGTTCCAACTTCGGATT
	SKIL	FW:GGTTGCCCCAAATGTGTCAC
		REV:CAGCAACAAGGCCAATTCCC
	SMAD7	FW:AGAGTGGGGAGGCTCTACTG
		REV:TCTGCACCAGCTGACTCTTG
	IL-2	FW:AACTCCTGTCTTGCATTGCAC
		REV:GCTCCAGTTGTAGCTGTGTTT
Mice	*Hprt1*	FW:CTGGTTAAGCAGTACAGCCCCAA
		REV:CAGGAGGTCCTTTTCACCAGC
	Hamp	FW:TTGCGATACCAATGCAGAAGA
		REV:GATGTGGCTCTAGGCTATGTT
	Id1	FW:ACCCTGAACGGCGAGATCA
		REV:TCGTCGGCTGGAACACATG

Supplementary Figures

Supplementary Figure 1

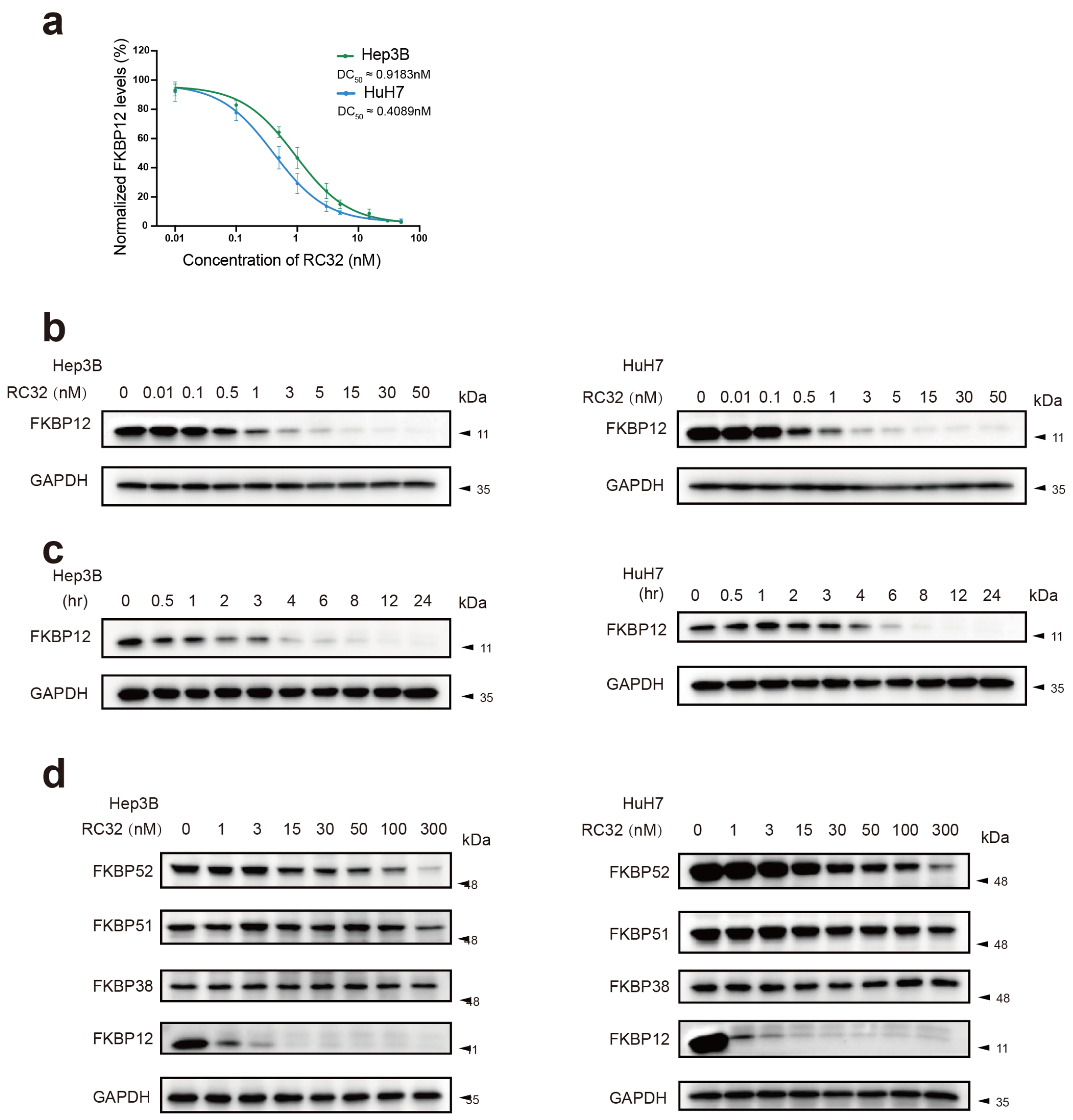

Supplementary Fig. 1 | RC32 induced efficient and specific FKBP12 degradation in hepatocellular carcinoma cells. (a) DC_{50} of RC32 in hepatocellular carcinoma cell lines. Hep3B and HuH7 were treated with indicated concentrations of RC32 for 12 hrs and their FKBP12 protein levels were quantified by Western Blots and grayscale analysis. (b) Hep3B or HuH7 cells were treated with

indicated concentrations of RC32 for 12 hrs and FKBP12 protein levels were monitored by Western Blotting. GAPDH served as a loading control. (c) Hep3B or HuH7 cells were treated with 15 nM RC32 for indicated time periods and FKBP12 protein levels were monitored by Western Blotting. (d) Hep3B or Huh7 cells were treated with indicated concentration of RC32 for 12 hrs, followed by Western Blot analysis of FKBP51, FKBP52, FKBP38 and FKBP12 proteins. GAPDH served as loading control.

Supplementary Figure 2

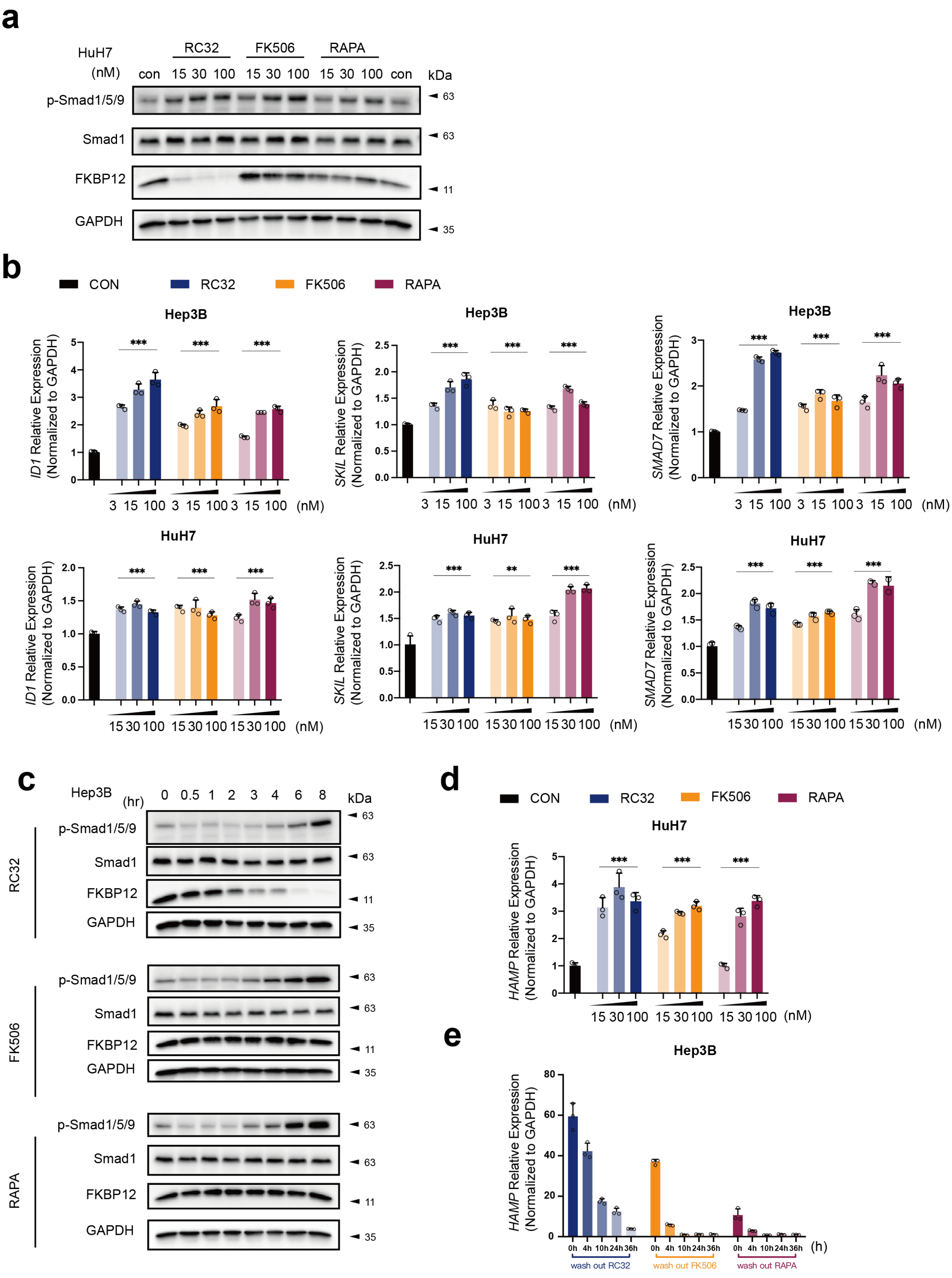

Supplementary Fig. 2 | RC32 elevated BMP signaling and Hepcidin expression in hepatocellular carcinoma cells. (a) HuH7 cells were treated with 3, 15, 100 nM of RC32, FK506 or Rapamycin for

15 hrs. BMP signaling was verified by phosphorylation of Smad1 (Ser463/465)/ Smad5 (Ser463/465)/ Smad8 (Ser465/467). Total Smad1 and GAPDH were used as loading controls. (b) Hep3B or HuH7 cells were treated with drugs for 15 hrs and then mRNA levels of BMP target genes *ID1*, *SKIL*, *SMAD7* were quantified by RT-qPCR. The results are presented as the mean ± SD, n = 3. one-way analysis of variance (ANOVA), *p < 0.05, **p ≤ 0.01, ***p ≤ 0.001. (c) Time course of RC32/FKB506/Rapamycin induced BMP activation in Hep3B cells. Cells were treated with 15 nM drugs for indicated time periods. FKBP12 degradation and BMP activation (p-Smad1/5/8) were detected by Western Blotting. Total Smad1 and GAPDH were used as loading controls. (d) HuH7 cells were treated with drugs as indicated concentrations for 15 hrs and then collected for RT-qPCR analysis of hepcidin (*HAMP*) expresison. The results are presented as the mean ± SD, n = 3. one-way analysis of variance (ANOVA), *p < 0.05, **p ≤ 0.01, ***p ≤ 0.001. (e) Duration of enhanced Hepcidin expression after RC32, FK506 or Rapamycin withdrawal. Hep3B cells were treated with 15 nM drugs for 15 hrs and then further cultured without drugs for indicated time periods. Hepcidin expression were quantified by RT-qPCR.

Supplementary Figure 3

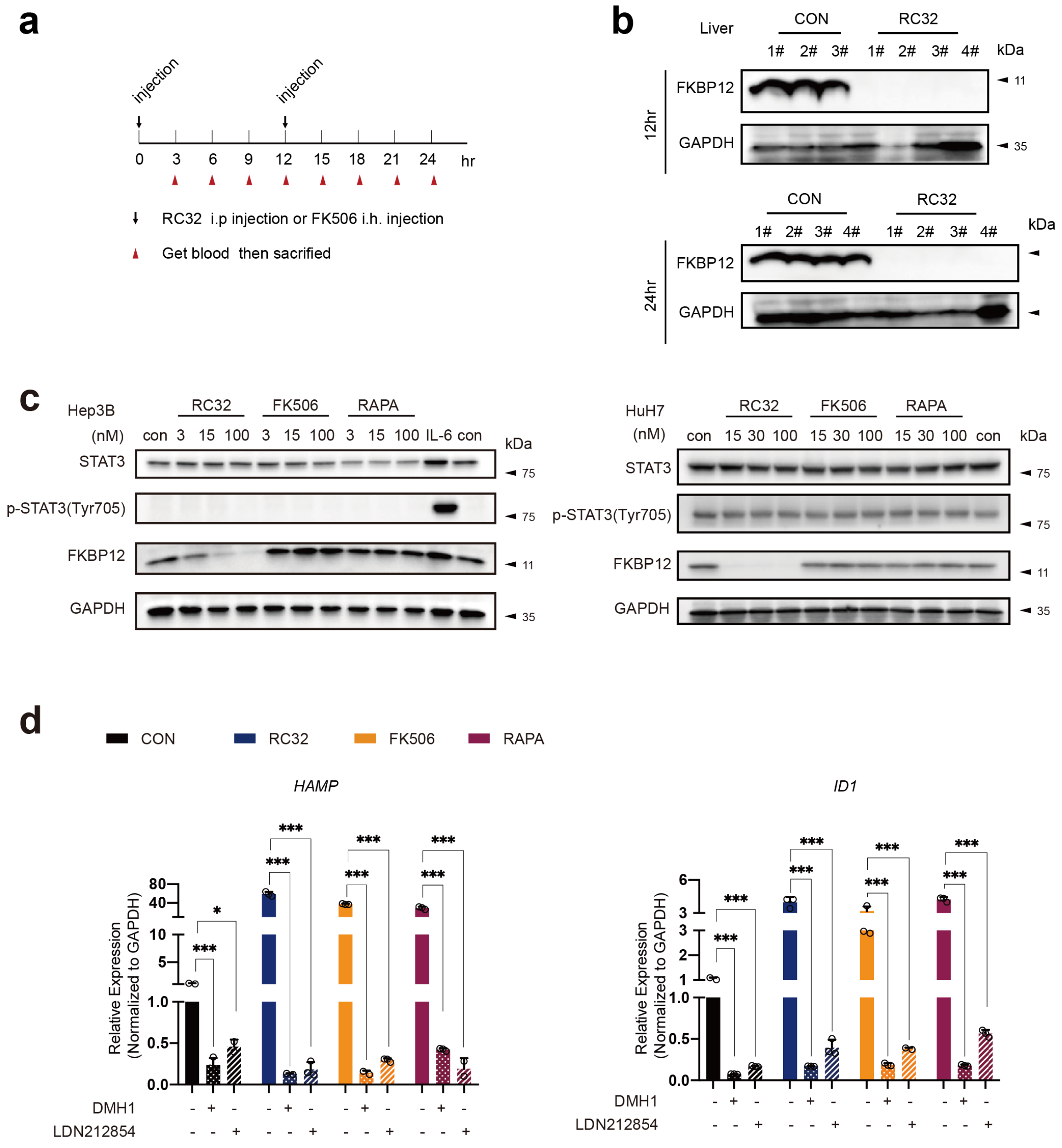

Supplementary Fig. 3 | RC32 elevated Hepcidin expression through BMP pathway. (a) Schematic description of experiment design. Wild-type (WT) C57BL/6N male mice (8 weeks old) were used with 4-6 mice for each sample. For FK506 treatment, mice were injected with FK506 or dissolvent at 0 and 12 hr via subcutaneous injections and sacrificed at indicated time points. For RC32 treatment, mice

were treated with RC32 or dissolvent at 0 and 12 hr by intraperitoneal injections and sacrificed at indicated time points. Dissolvent (vehicle) group were sacrificed at 3 and 15hr. (b) Liver tissues were collected from control mice or mice given RC32 (30 mg/kg, i.p.) for 12 hrs or 24 hrs. Tissue lysates were analyzed by Western Blotting for FKBP12 degradation. GAPDH served as loading controls. (c) Hep3B or HuH7 cells were treated with 3, 15, 100 nM RC32, FK506 or Rapamycin for 15 hrs and IL-6 (20 ng) was used as a positive control. JAK/STAT3 signaling activation was detected by phosphorylation of STAT3 (Tyr705, p-STAT3) in Western Blotting. Total STAT3 and GAPDH were used as loading controls. (d) Hep3B cells pretreated with ALK2 inhibitors DMH1 (500 ng/mL) or LDN212854 (150 ng/mL) for 5 hrs were then treated with 15 nM RC32, FK506 or rapamycin for 15 hrs. mRNA levels of *Hepcidin* (HAMP) and BMP target gene *ID1* were verified by RT-qPCR. The results are presented as the mean ± SD, n = 3. one-way analysis of variance (ANOVA), *p < 0.05, **p ≤ 0.01, ***p ≤ 0.001.

Supplementary Figure 4

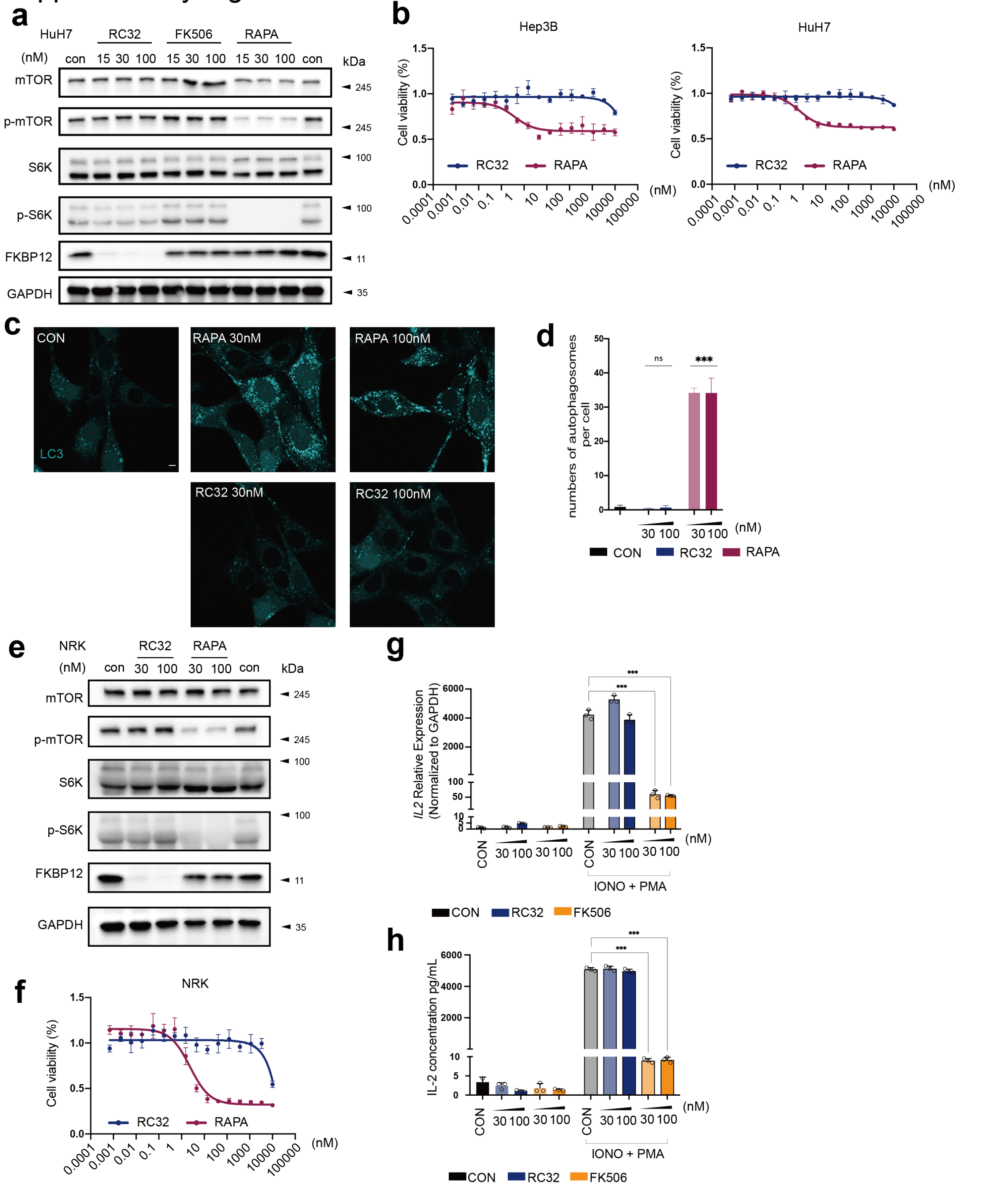

Supplementary Fig. 4 | RC32 did not inhibit mTOR activity or Calcineurin/NFAT pathway. (a) HuH7 cells were treated with 15, 30 100 nM of RC32, FK506 or Rapamycin for 15 hrs and then

subjected to Western Blotting. mTOR activity was detected by phosphorylation of mTOR (Ser2448, p-mTOR) and phosphorylation S6 Kinase (Thr389, p-S6K). Total mTOR, S6 Kinase (S6K) and GAPDH were used as loading controls. (b) Hep3B, Huh7 in 96-well plates were treated as indicated for 3 days and cell viability was measured using CCK8. The results are presented as the mean ± SD, n = 3. (c and d) Autophagy induction. NRK-LC3-CFP cells were treated for 15 hrs with drugs as indicated and fluorescence images were presented. Scale bars in (c) is 5 μm. (d) Numbers of LC3 puncta were counted from three independent experiments in each at least 100 cells were analyzed using Imaris. Error bars indicate the Mean-S.D. (e) NRK-LC3-CFP cells were treated with drugs as indicated for 15 hrs and subjected to Western Blotting. (f) NRK-LC3-CFP cells in 96-well plates were treated as indicated for 3 days and cell viability was measured using CCK8. The results are presented as the mean ± SD, n = 3. (g) Jurkat cells were pretreated with RC32 or FK506 for 4 hrs and then stimulation with ionomycin/PMA for 24 hrs. mRNA levels of *IL-2* were quantified by RT-qPCR and normalized to *GAPDH.* The results are presented as the mean ± SD, n = 3. one-way analysis of variance (ANOVA), *$p < 0.05$, **$p \leq 0.01$, ***$p \leq 0.001$. (h) IL-2 concentration in culture medium released by Jurkat cells after ionomycin/PMA stimulation were measured using IL-2 ELISA kit. The results are presented as the mean ± SD, n = 3. one-way analysis of variance (ANOVA), *$p < 0.05$, **$p \leq 0.01$, ***$p \leq 0.001$.

Supplementary Figure 5

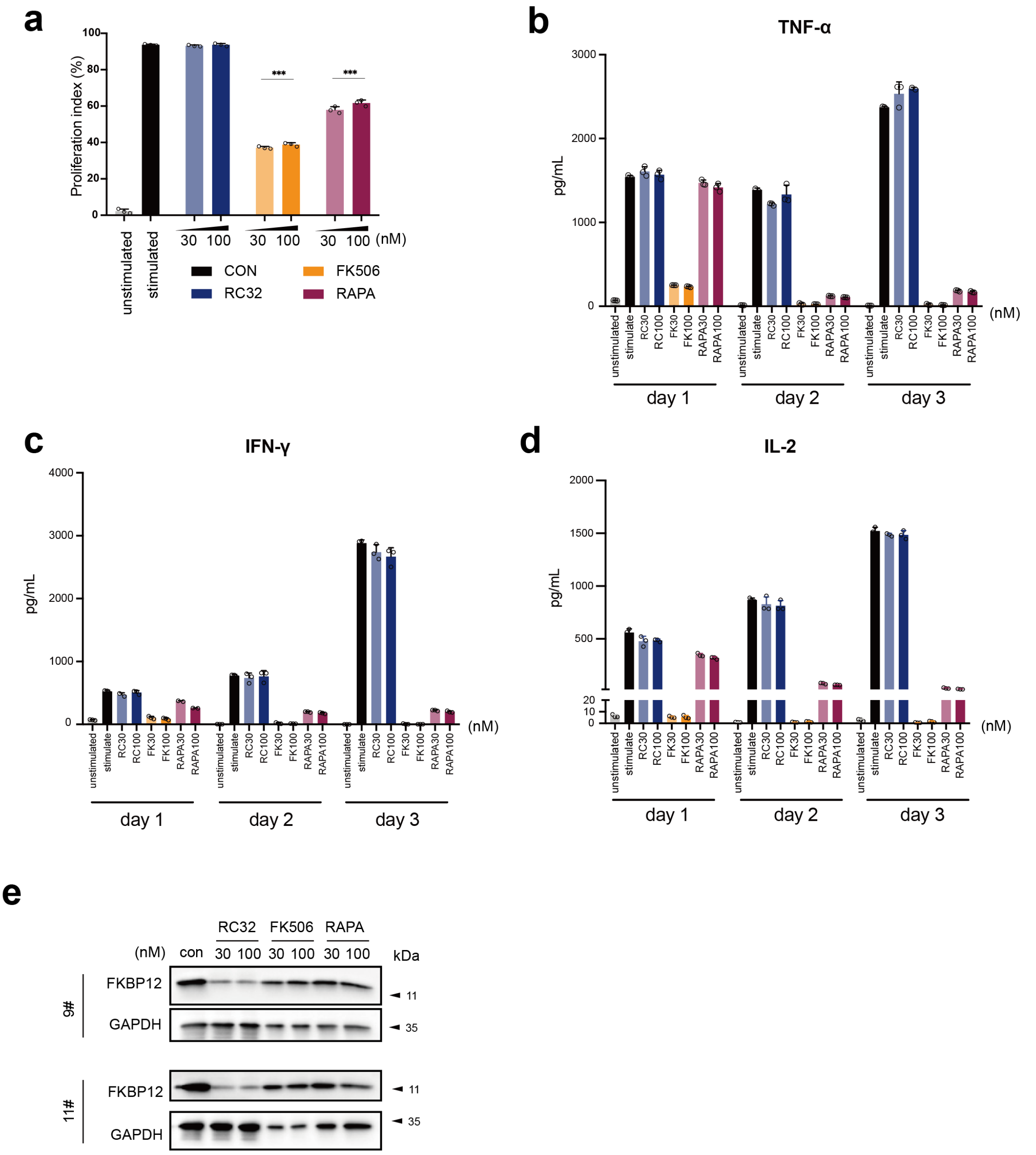

Supplementary Fig. 5 | RC32 did not inhibit PBMC proliferation *in vitro*. (a) Proliferation index of Fig. 1h is calculated and presented as the mean ± SD, n = 3. one-way analysis of variance (ANOVA), *p < 0.05, **p ≤ 0.01, ***p ≤ 0.001 **** p ≤ 0.0001. (b-d) Cytokine secretion during PBMC

stimulation. Culture medium from CD3/CD28 stimulated PBMC was collected and TNF-α, IFN-γ and IL-2 concentration determined by ELISA assay. RC32, FK506 or Rapamycin was added as indicated. Culture medium was replaced every day and measurement performed 1, 2 and 3 days after stimulation. The results are presented as the mean ± SD, n = 3. (e) RC32 induced FKBP12 degradation in PBMC. PBMCs from two donors after the stimulated proliferation assay (description in **Fig. 1h**) were subjected to Western Blotting analysis for FKBP12 degradation. GAPDH served as loading control.
